# Supplementary figures and images for: An adhesion signaling axis involving Dystroglycan, β1-Integrin, and Cas adaptor proteins regulates the establishment of the cortical glial scaffold
Source: PLoS Biol. 2023 Aug 4;21(8):e3002212. doi: 10.1371/journal.pbio.3002212 (PMC10431685; doi:10.1371/journal.pbio.3002212)

**A****WT****Chicken anti-EGFP****EGFP Tbr1 DAPI****EGFP**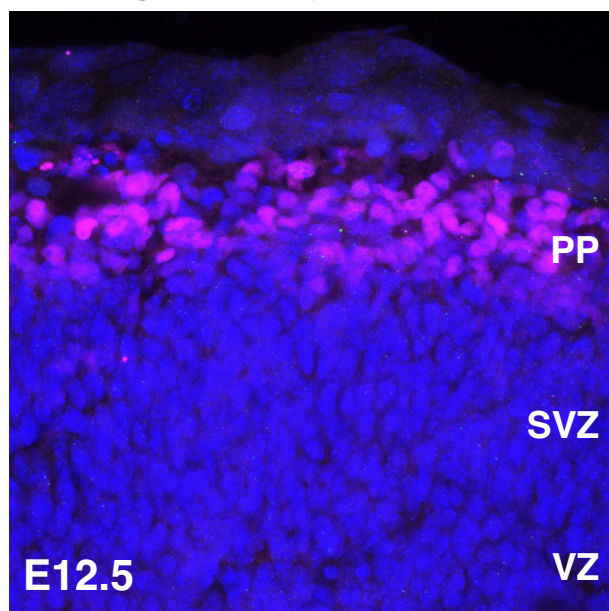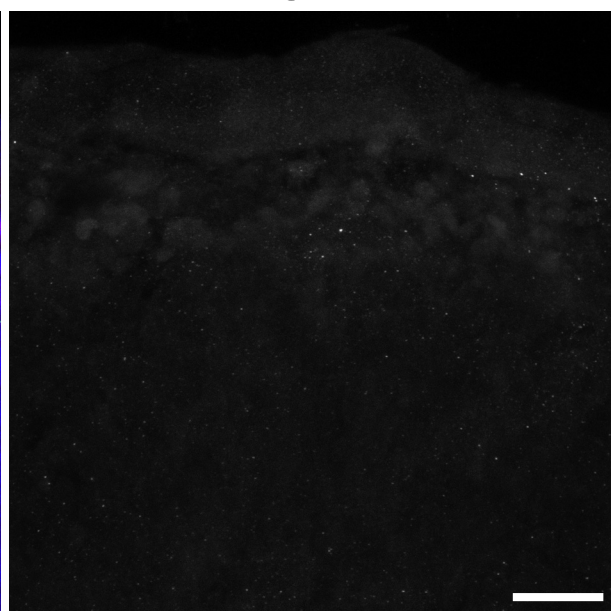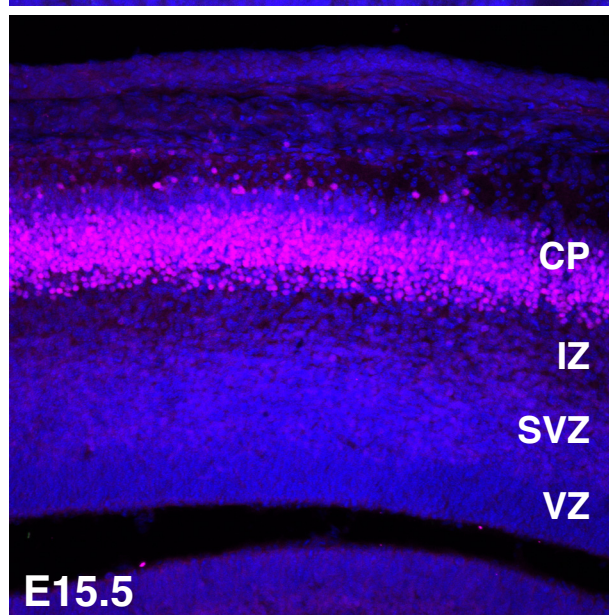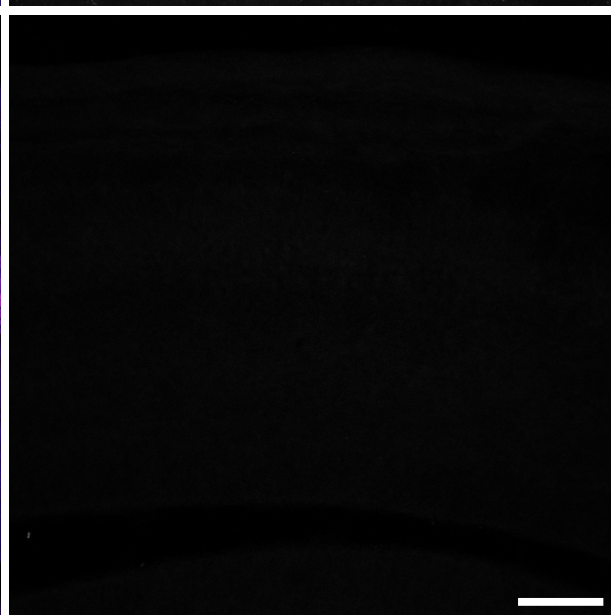**B****Rabbit anti-EGFP****EGFP Nestin DAPI****EGFP**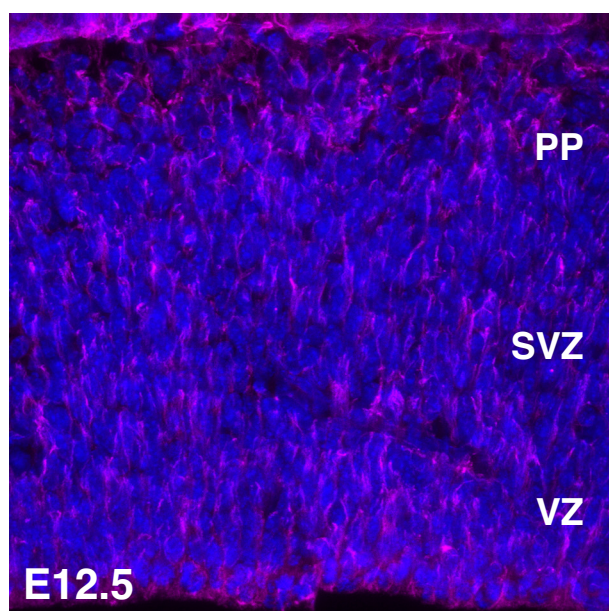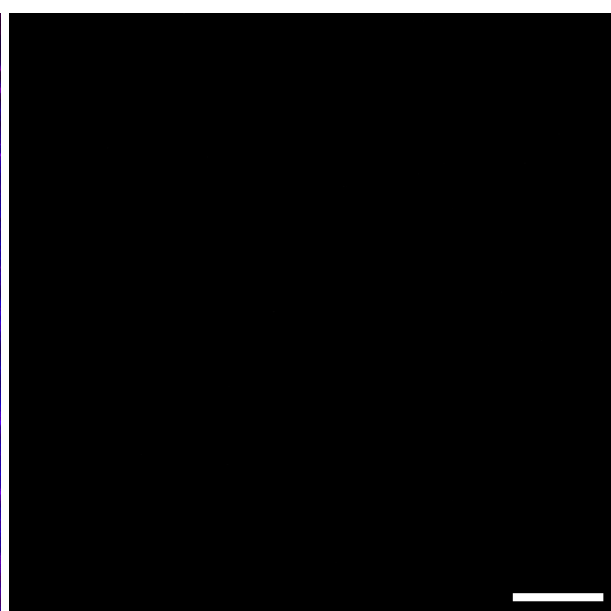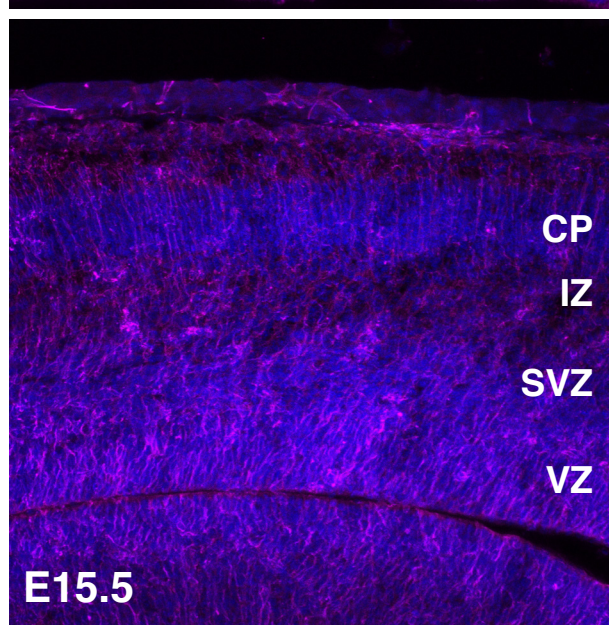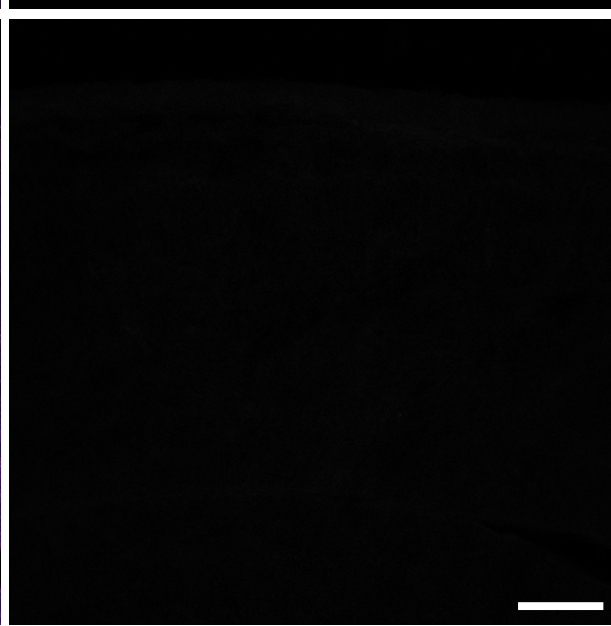

Supplement: S1 Fig — (A, B) Immunostaining of cortical sections with the same chicken-anti EGFP (A) and rabbit-anti EGFP (B) antibodies used to detect it in p130Cas::BacEGFP animals produce negligible signal on WT sections at E12.5 and E15.5. Magenta channel in (A) is Tbr1 and in (B) is Nestin. Dapi counterstain is shown in blue. n = 3 animals. Scale bars for A and B (top): 25 μm; A and B (bottom): 75 μm. CP, cortical plate; EGFP, enhanced green fluorescent protein; IZ, intermediate zone; PP, preplate, SVZ, subventricular zone; VZ, ventricular zone; WT, wild-type. (PDF) [file pbio.3002212.s001.pdf]

**A***Emx1Cre;Ai14***tdTomato**  
**DAPI**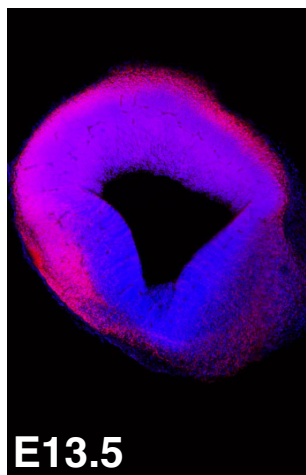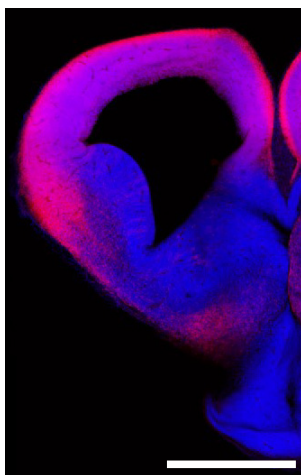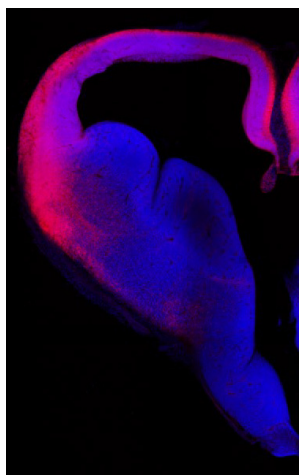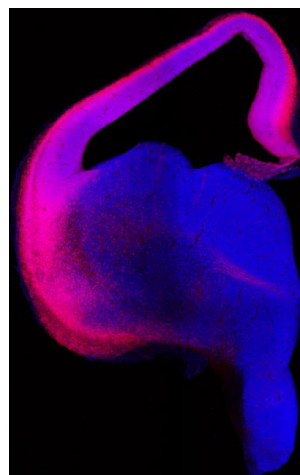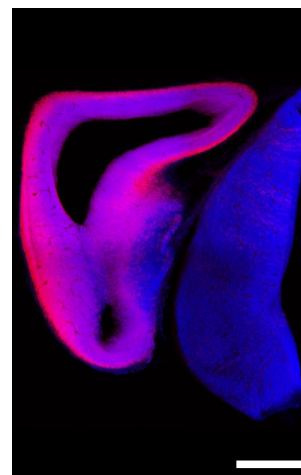**B***p130Cas**CasL Sin**p130Cas**CasL**Sin***Control**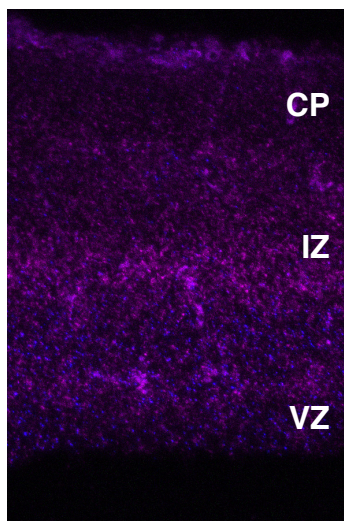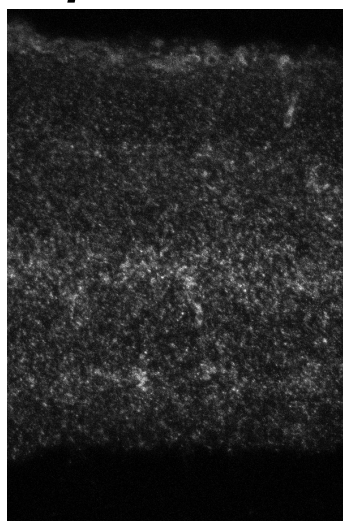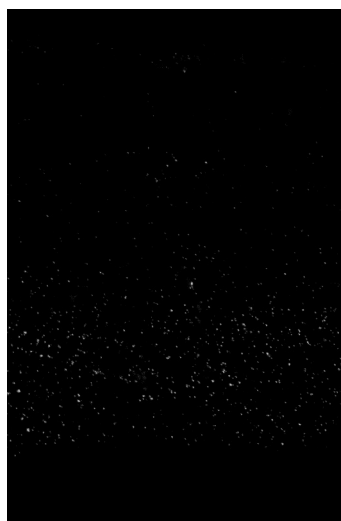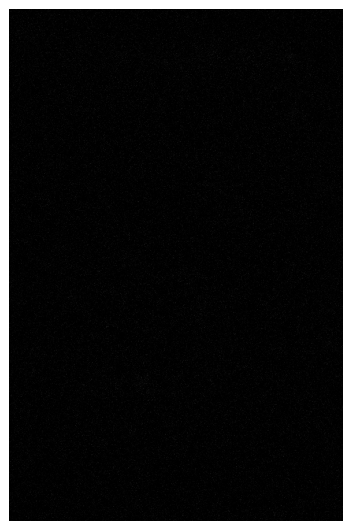***Emx1Cre;CasTcKO***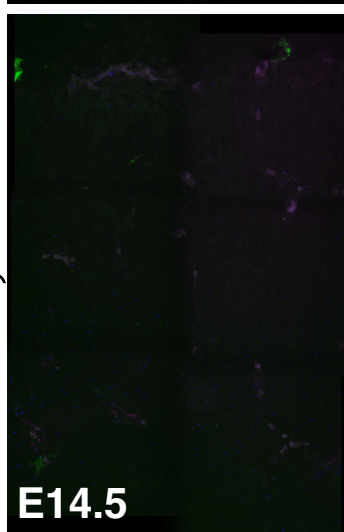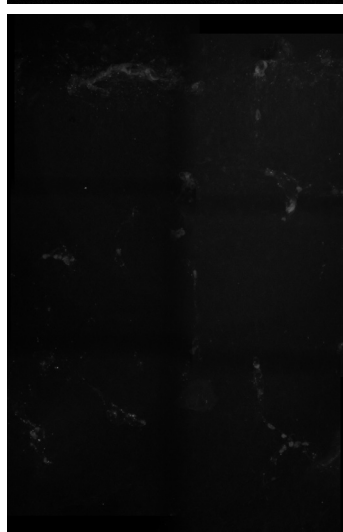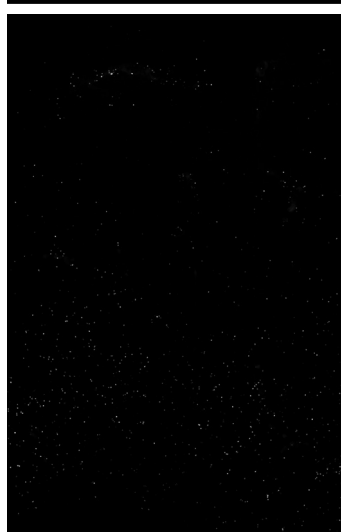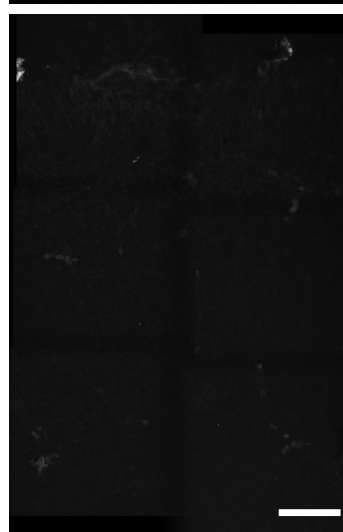

Supplement: S2 Fig — (A) tdTomato staining of E13.5 Emx1Cre;Ai14 coronal cortical sections. Emx1Cre drives Cre recombinase expression in cortical neural progenitors and RGCs that generate excitatory neurons in the forebrain and hippocampus. (B) RNAscope at E14.5 shows that Emx1Cre;CasTcKO cortices do not produce functional p130Cas transcripts. Control littermates express p130Cas transcripts strongly in the IZ and broadly throughout the neocortex. Neither controls nor Emx1Cre;CasTcKO animals express Sin or CasL. n = 3 animals per genotype. Scale bars for A: 500 μm; D: 100 μm. CP, cortical plate; IZ, intermediate zone; RGC, radial glial cell; SVZ, subventricular zone; VZ, ventricular zone. (PDF) [file pbio.3002212.s002.pdf]

**A****Control*****Emx1Cre;CasTcKO*****Cux1 Ctip2 DAPI**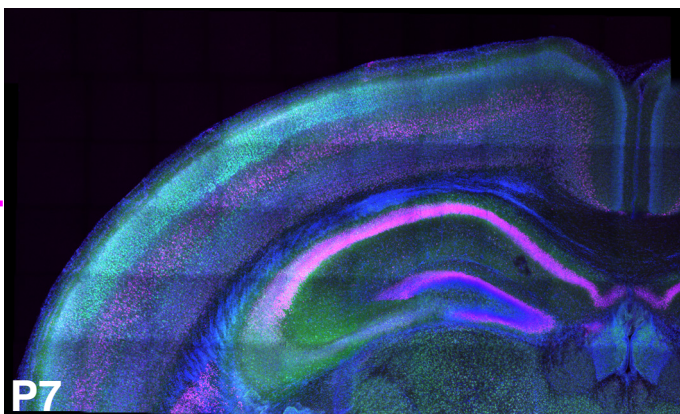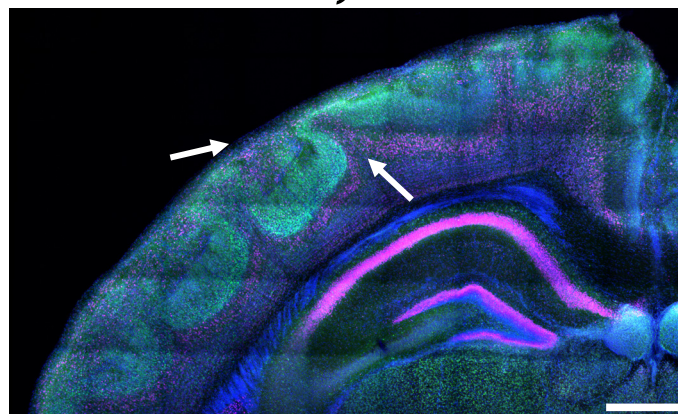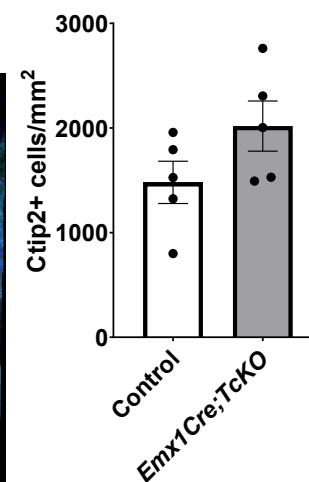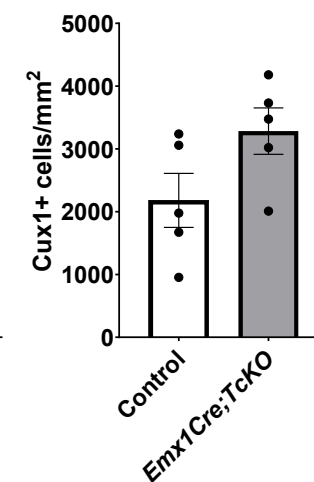**B****Control*****Emx1Cre;CasTcKO*****Rorβ DAPI**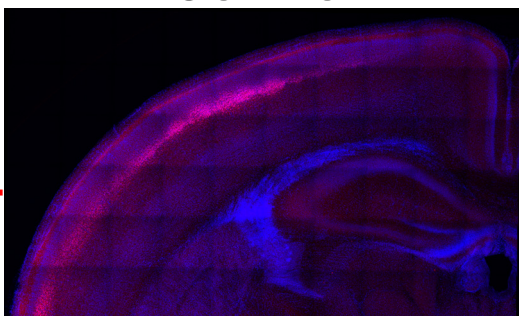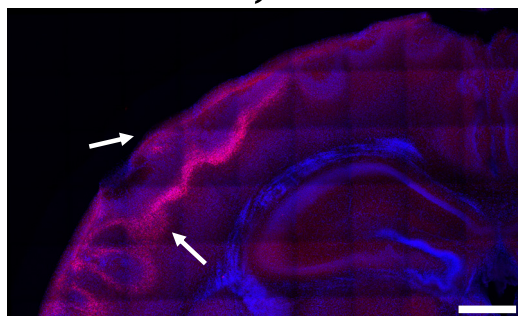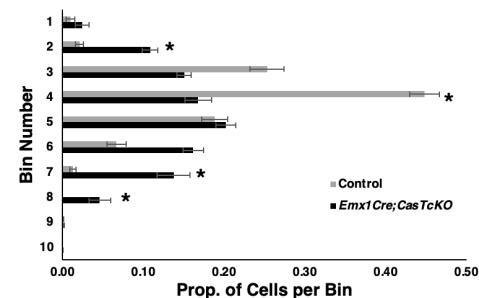**C****Tbr1 DAPI**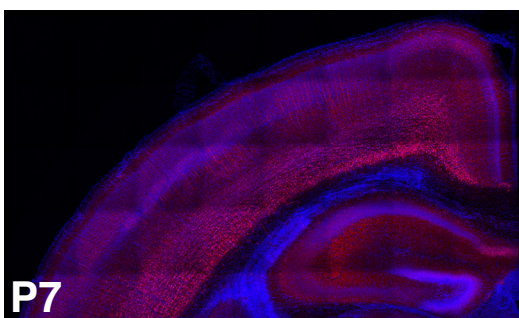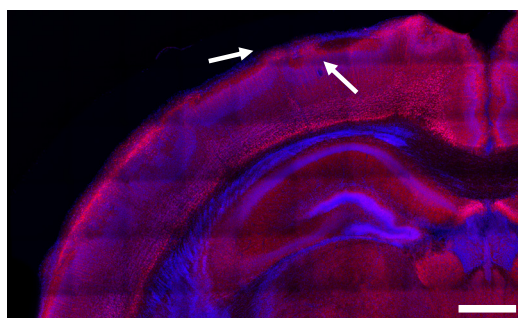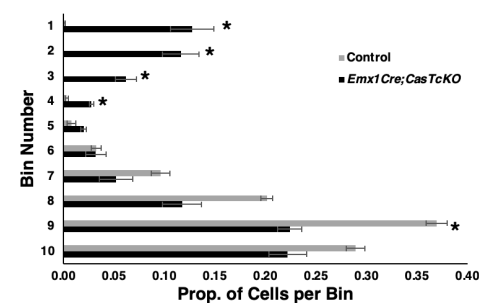

Supplement: S3 Fig — (A-C) Immunostaining of P7 control and Emx1Cre;CasTcKO coronal sections using the layer markers Cux1 (green, layer II/III) and Ctip2 (magenta, layers V and VI) (A), Rorβ+ (B, red, layer IV), and Tbr1+ (C, red, layer VI). Neurons appear misplaced in Emx1Cre;CasTcKO mice (white arrows). DAPI (blue) was used to counterstain. (A) Right panels: quantification of Ctip+ and Cux1+ neuron density. Two-tailed t test ns: p = 0.12 for Ctip2+ cells; p = 0.09 for Cux1+ cells; n = 5 independent animals for each genotype, 3 sections per animal. (B, C) Right panels: quantification of the proportion of Rorβ+ and Tbr1+ cells per bin. Bin 1 is the MZ and bin 10 is ventral to layer VI. n = 3 animals per group, 3 sections per animal, Mann–Whitney U test and Bonferroni correction, *p < 0.005. Values given are mean ± SEM. For data plotted in graphs, see S2 Data. Scale bars: 500 μm. (PDF) [file pbio.3002212.s003.pdf]

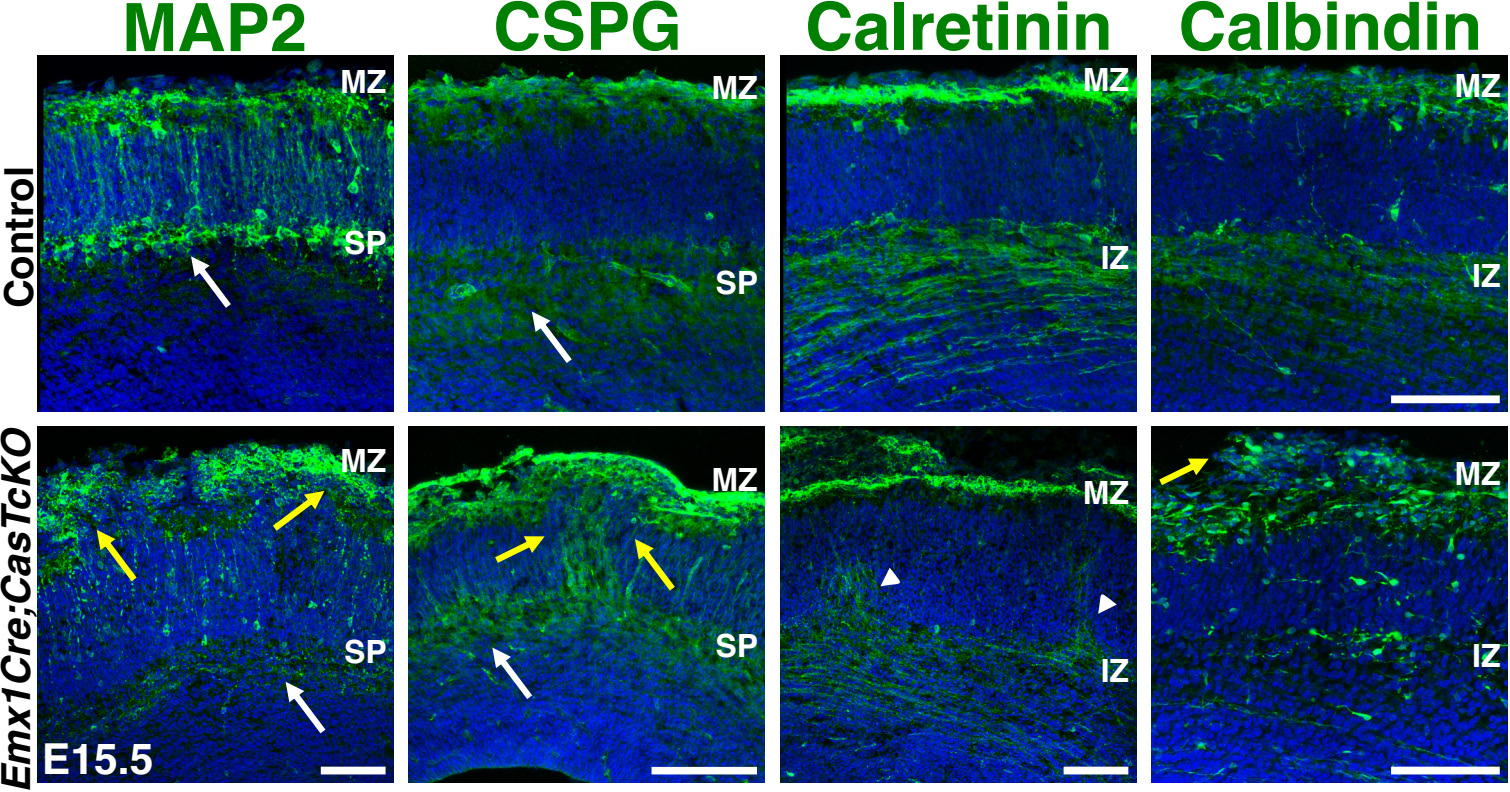

Supplement: S5 Fig — Immunostaining of control and Emx1Cre;CasTcKO coronal sections for MZ and SP (white arrows) markers at E15.5. MAP2, CSPG, Calretinin, and Calbindin show clustering of cells (yellow arrows). CSPG+ cells form a column connecting the SP with the MZ (yellow arrow). Thalamocortical afferents labeled with Calretinin show ectopic bundles extending from the IZ (white arrowheads). DAPI was used as a counterstain (blue). n = 3 animals per genotype. Scale bars: 100 μm. IZ, intermediate zone; MZ, marginal zone; SP, subplate. (PDF) [file pbio.3002212.s005.pdf]

# A

## Control

## *Emx1Cre;CasTcKO*

# B

# C

# D

aCasp3 DAPI

aCasp3 DAPI

aCasp3 DAPI

aCasp3 DAPI

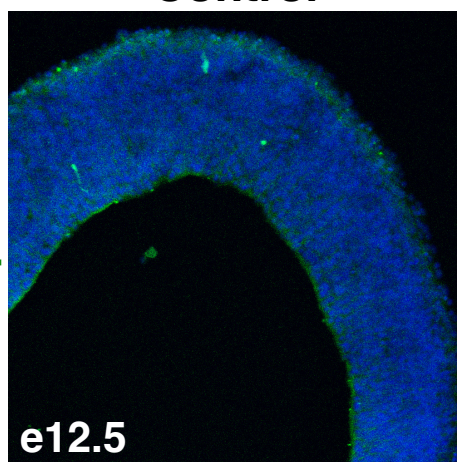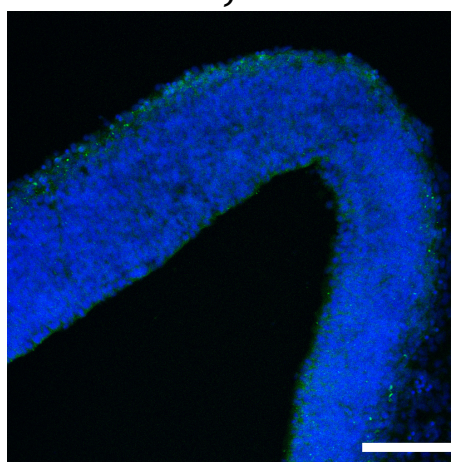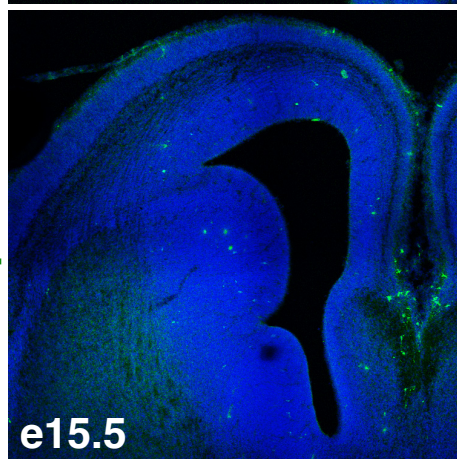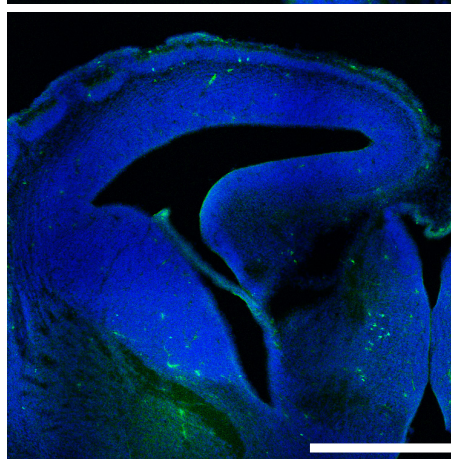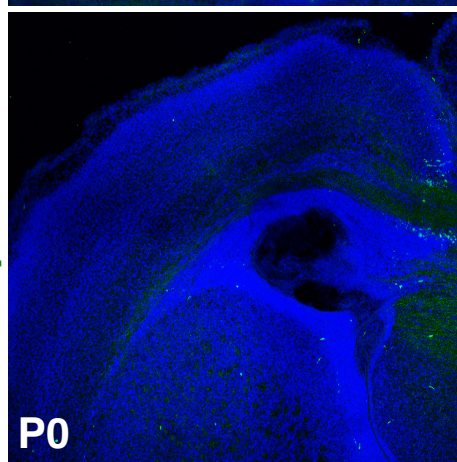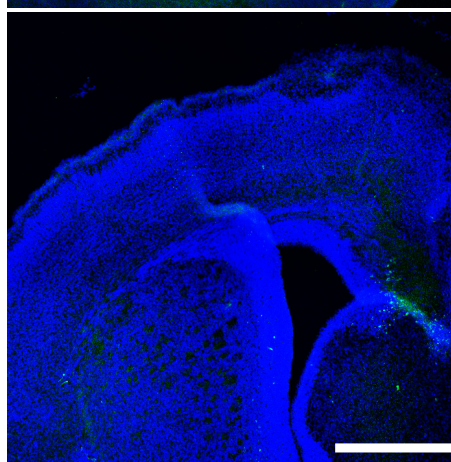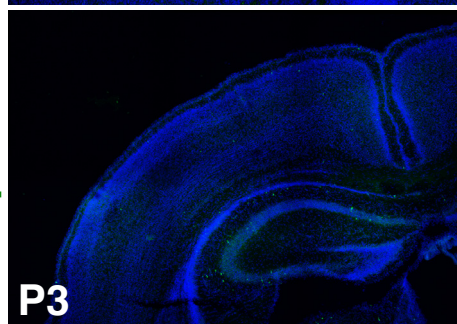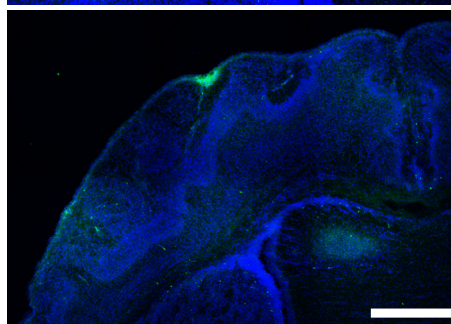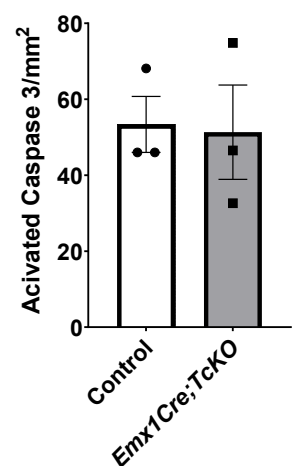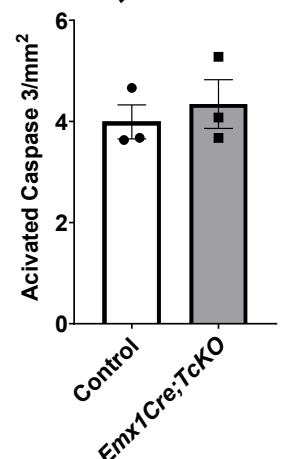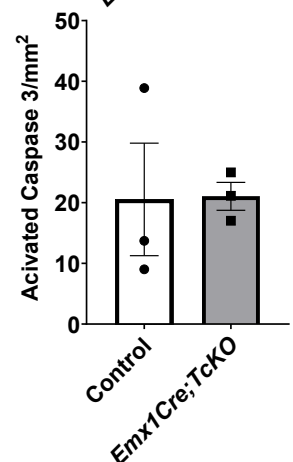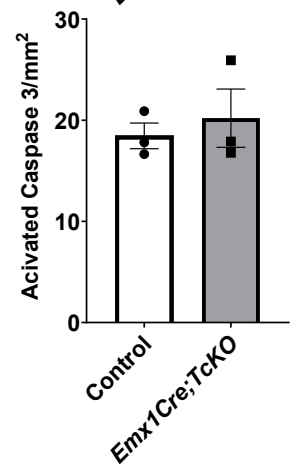

Supplement: S6 Fig — (A-D) Immunostaining for activated Caspase3 (aCasp3, green) on E12.5 (A), E15.5 (B), P0 (C), or P3 (D) coronal sections of control and Emx1Cre;CasTcKO cortices. Sections were counterstained with DAPI (blue). No notable differences are observed between genotypes. Right panels: quantification of density of activated Caspase3. Values given are mean ± SEM, n = 3 independent samples per group, 3–5 sections per sample, Mann–Whitney U test and Bonferroni correction, ns for all stages (p ≥ 0.7). For data plotted in graphs, see S6 Data. Scale bars for A: 100 μm; B, C, D: 500 μm. (PDF) [file pbio.3002212.s006.pdf]

**A***NexCre;Ai14*tdTomato  
DAPI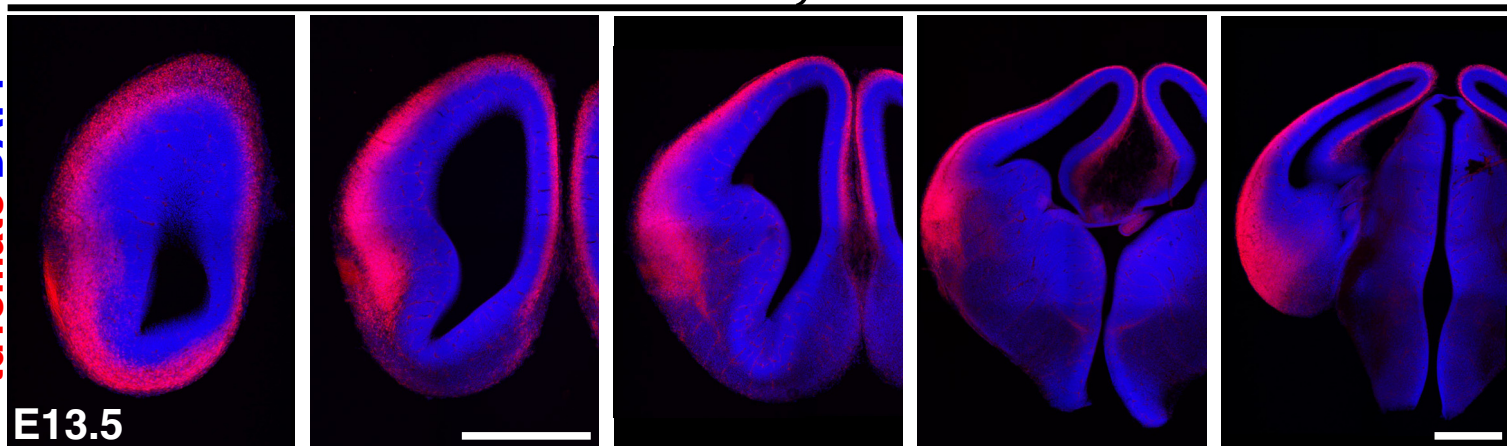**B**

Control

*NexCre;CasTcKO*DAPI  
*p130Cas*  
*CasL*  
*Sin*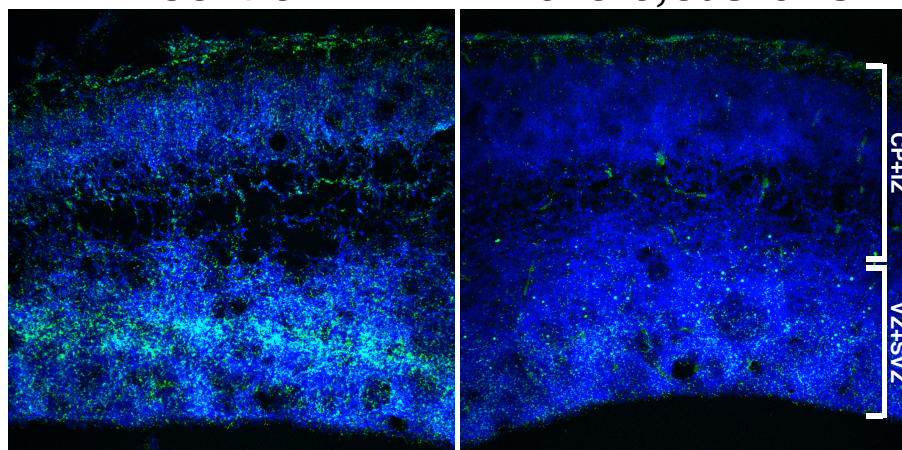*p130Cas**CasL**Sin*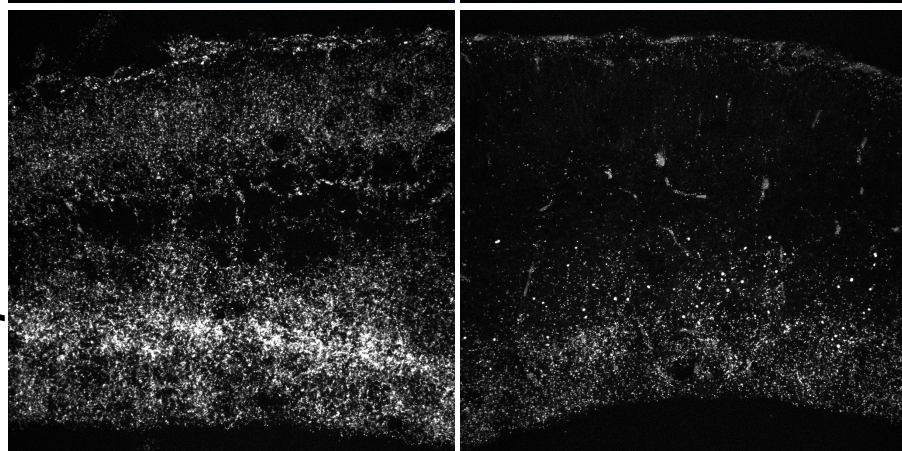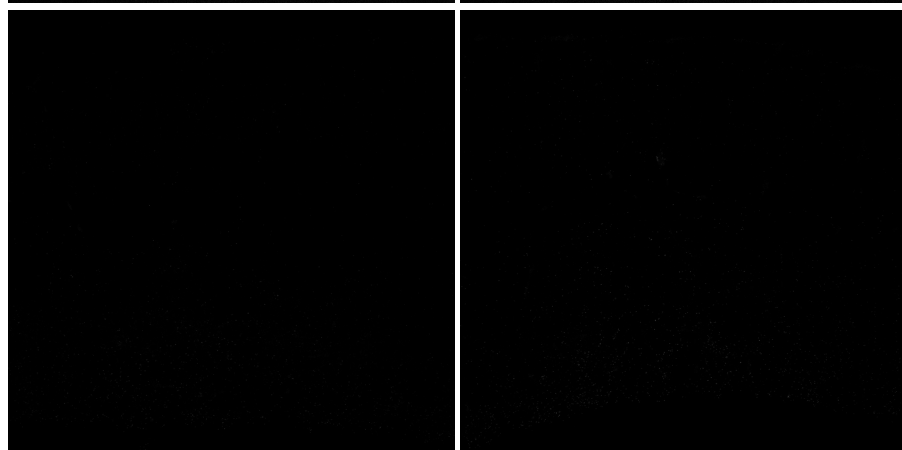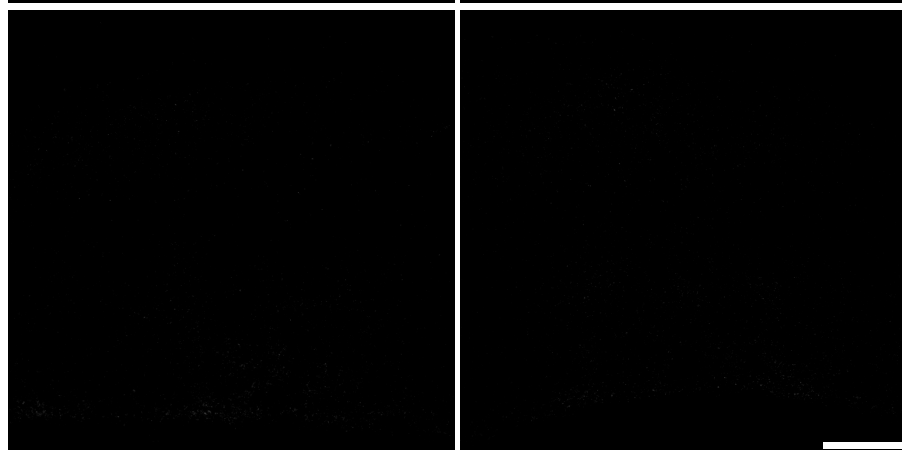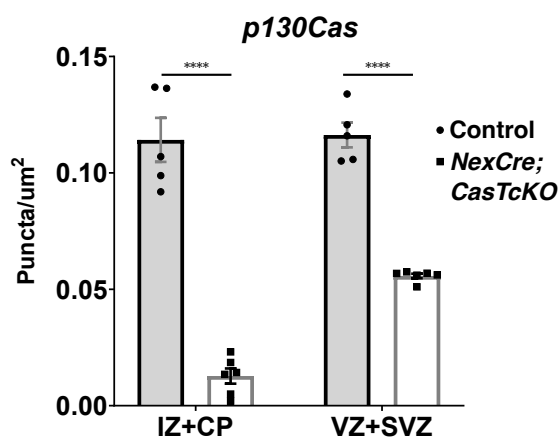

Supplement: S7 Fig — (A) tdTomato staining of E13.5 NexCre;Ai14 coronal cortical sections. NexCre targets postmitotic, premigratory neurons in the neocortex. (B) RNAscope at E16.5 indicates that NexCre;CasTcKO cortices express negligible levels of functional p130Cas transcripts in the IZ and CP and display significantly less transcript in the VZ and SVZ. Top right panel: quantification puncta per micron DAPI area in CP + IZ was 0.114 ± 0.009 for Nex-Cre+;CasTcKOflox/+ and 0.116 ± 0.005 for VZ + SVZ. For Nex-Cre+;CasTcKOflox/Δ, the average p130Cas puncta per micron DAPI area in CP + IZ was 0.013 ± 0.003 and 0.056 ± 001 for VZ and SVZ. Values given are mean ±SEM, n = 5–6, ***p < 0.0001 vs. control (one-way ANOVA; Tukey post hoc test). For data plotted in graphs, see S7 Data. Scale bars: 100 μm. CP, cortical plate; IZ, intermediate zone; SVZ, subventricular zone; VZ, ventricular zone. (PDF) [file pbio.3002212.s007.pdf]

**A****Control*****NexCre;CasTcKO*****Ror $\beta$  DAPI**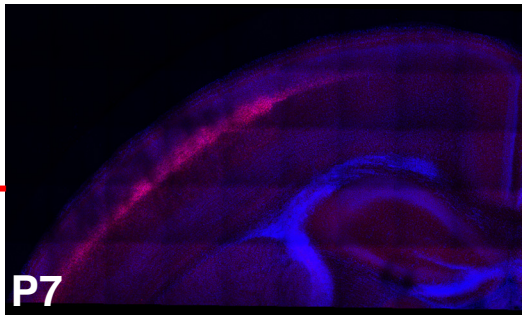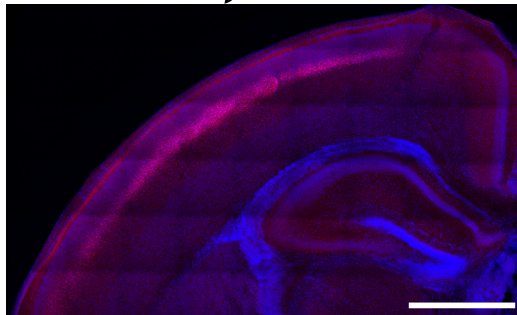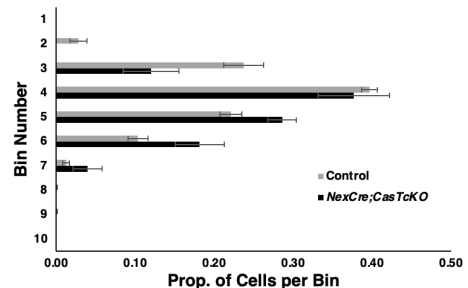**B****Tbr1 DAPI**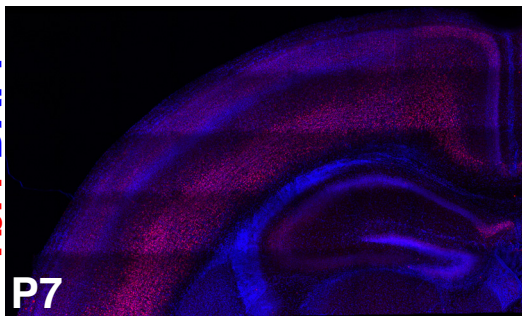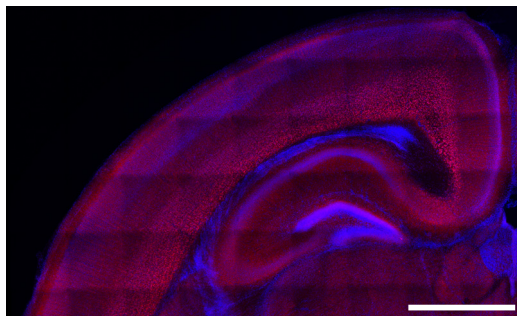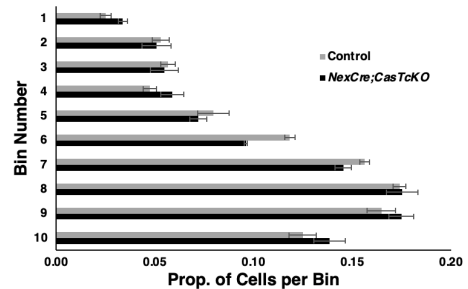

Supplement: S8 Fig — (A, B) Immunostaining of coronal sections for P7 control and NexCre;CasTcKO animals with layer makers Rorβ (A, red, layer IV) and Tbr1 (B, red, layer VI). DAPI was used to counterstain nuclei (blue). No overt lamination phenotypes are observed. Right panels: quantification of proportion of Rorβ+ (A) and Tbr1+ (B) cells per bin. Values given are mean ± SEM, n = 3 animals per group, 3 sections per animal, Mann–Whitney U test and Bonferroni correction, no significant differences (p > 0.05). For data plotted in graphs, see S9 Data. Scale bar: 500 μm. (PDF) [file pbio.3002212.s008.pdf]

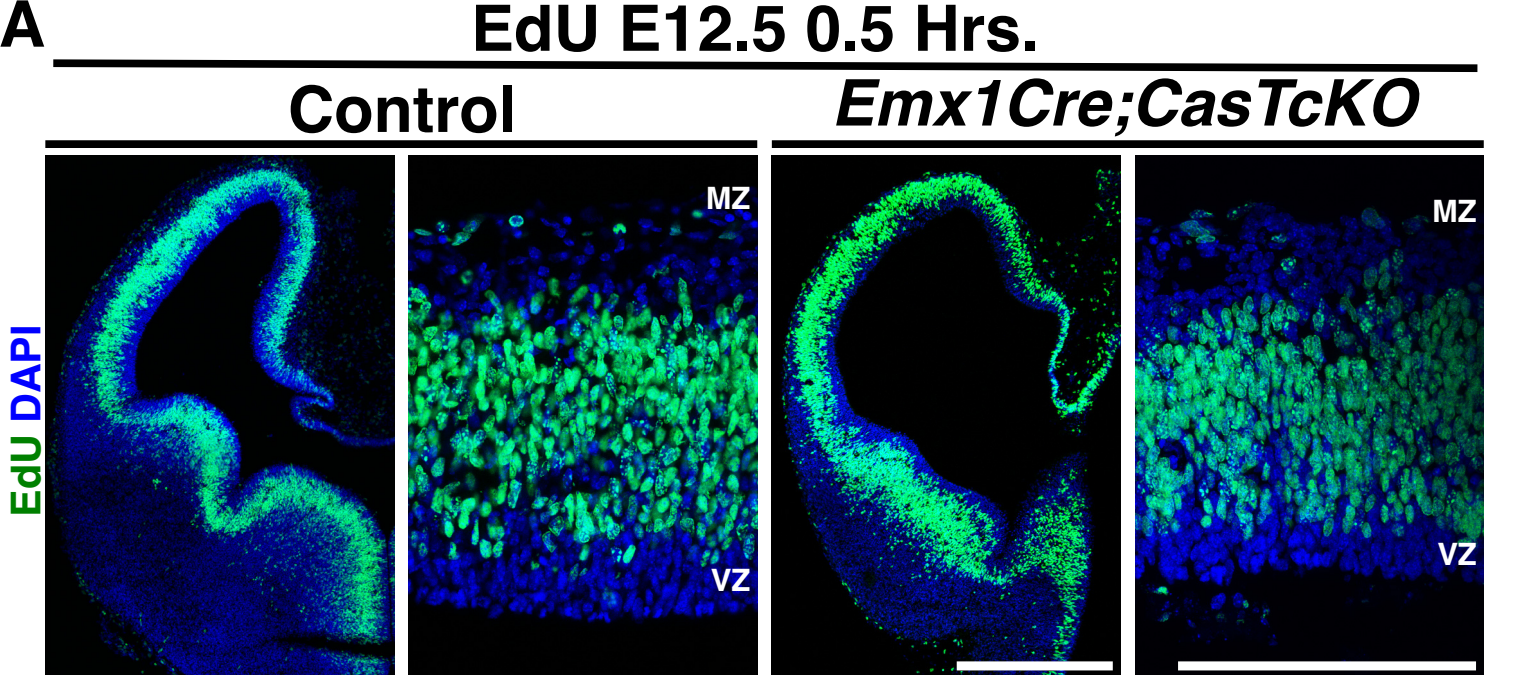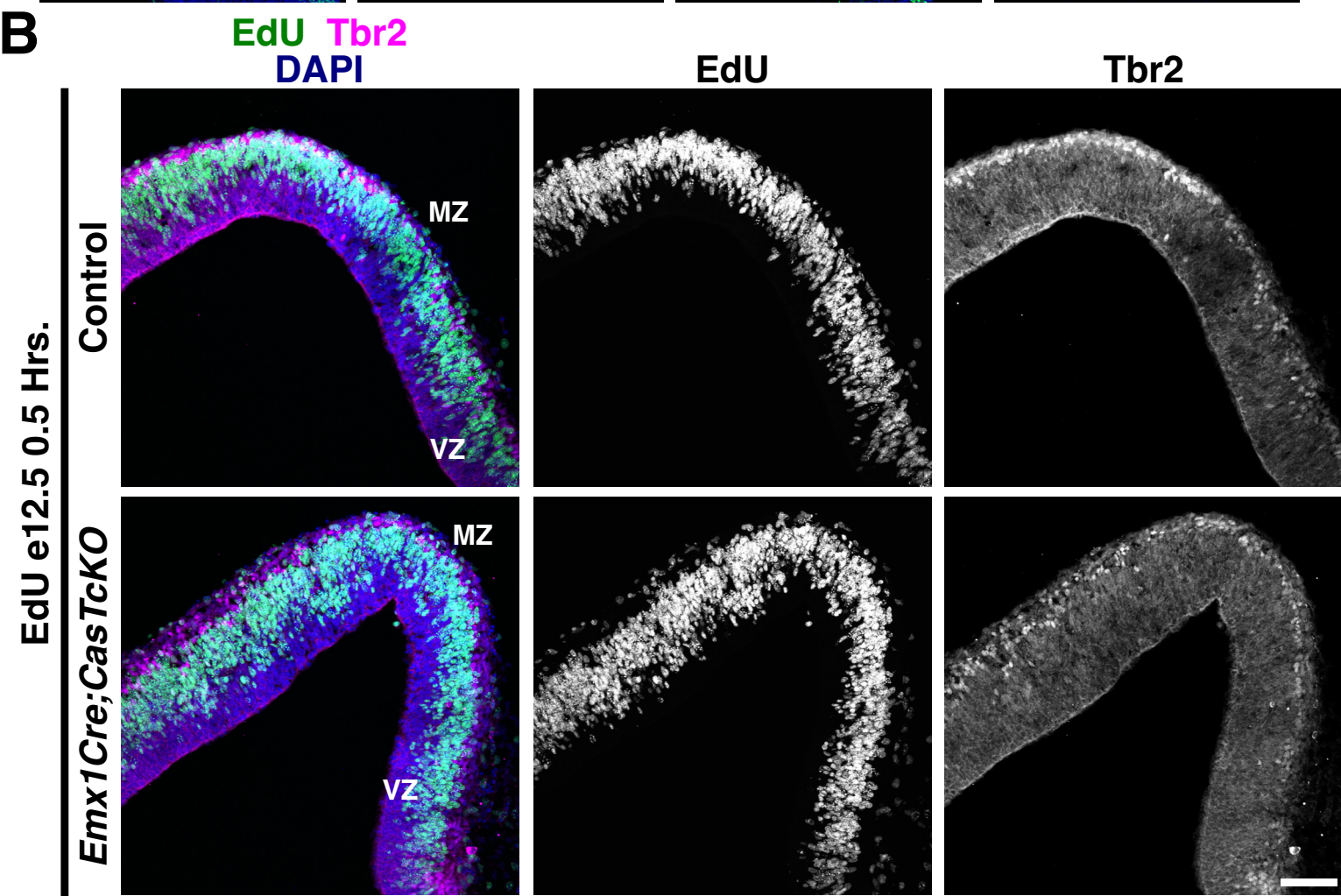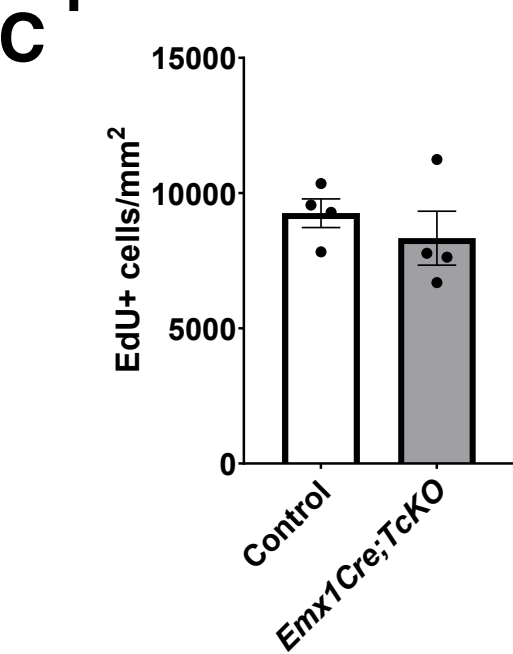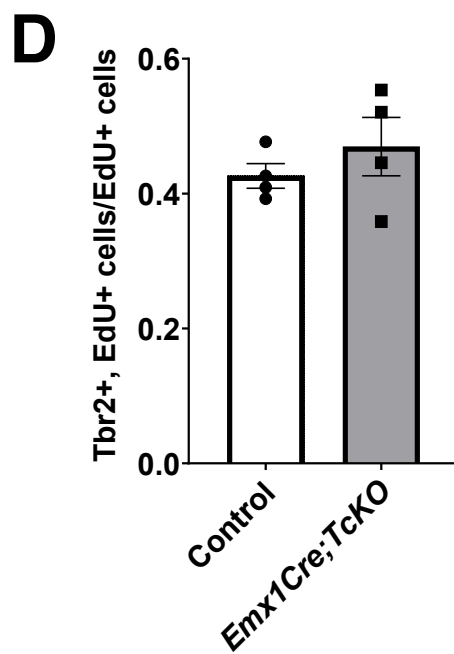

Supplement: S9 Fig — (A, B) Detection of proliferative cells in control and Emx1Cre;CasTcKO coronal sections using short-pulsed EdU at E12.5 (A, B, green), colabeled with the intermediate progenitor marker Tbr2 (B, magenta). Nuclei were counterstained with DAPI (blue). (C) Density of EdU+ cells/mm2 of cortex. p = 0.34, two-tailed Mann–Whitney U test, n = 4 independent samples per group, 3 sections per sample. (D) Proportion of Edu+ cells that coexpress Tbr2. p = 0.49, two-tailed Mann–Whitney U test, n = 4 independent samples per group, 2–3 sections per sample. Values given are mean ± SEM. For data plotted in graphs, see S11 Data. Scale bars: Lower magnification panel for A: 500 μm; Higher mag panels in A: 100 μm; B: 75 μm. (PDF) [file pbio.3002212.s009.pdf]

**A****Control*****NexCre;B1<sup>flox/flox</sup>*****Cux1 Ctip2 DAPI**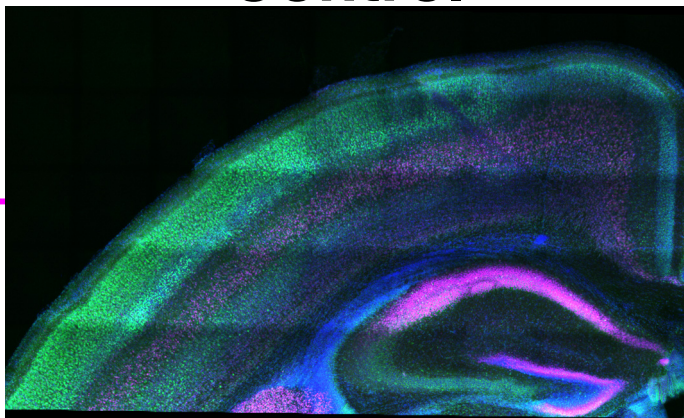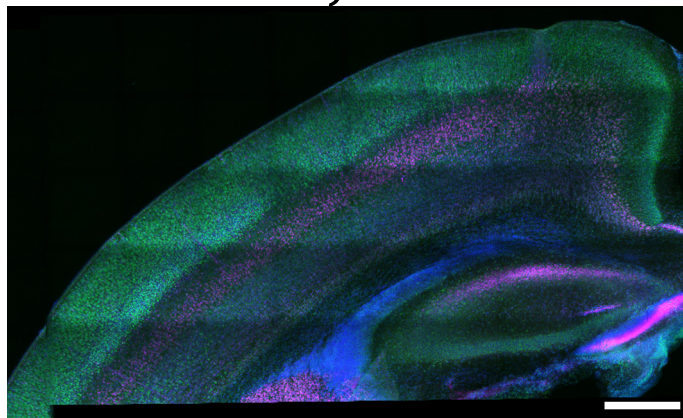**B****Rorb DAPI**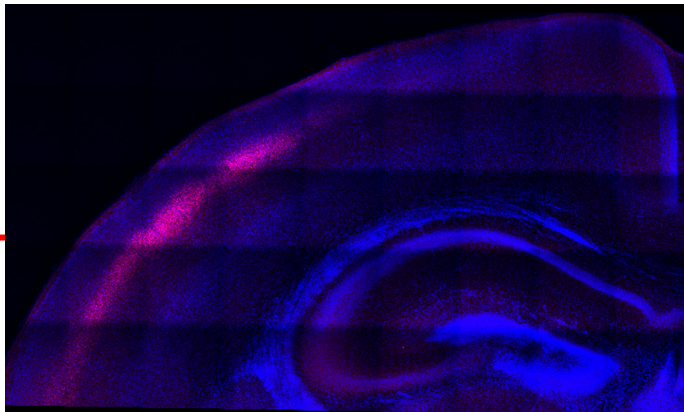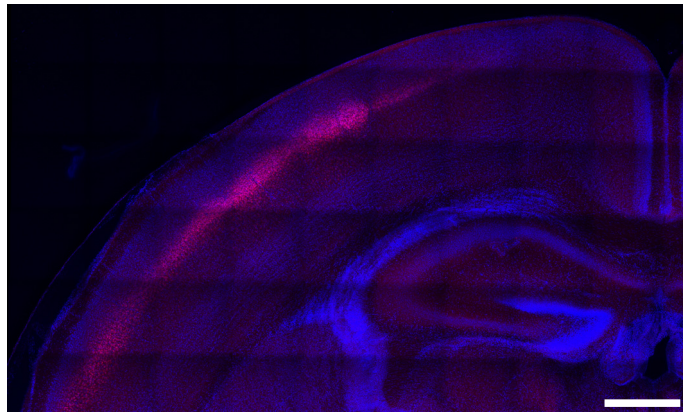**C****Tbr1 DAPI**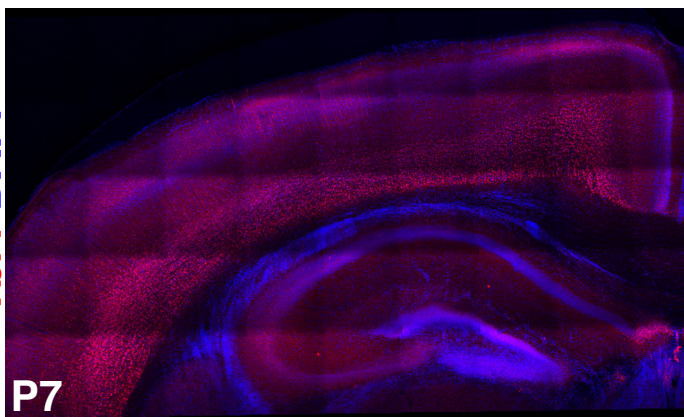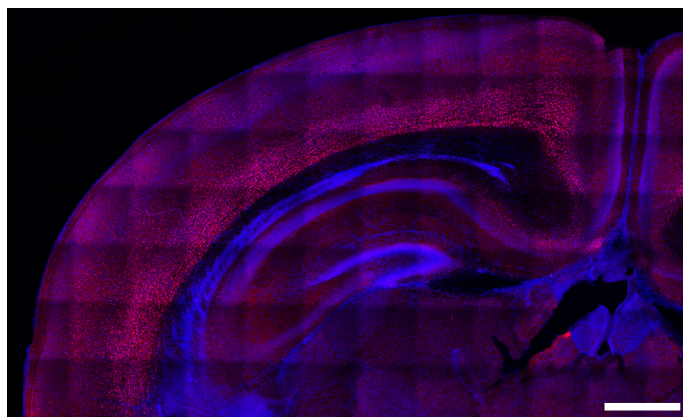**P7**

Supplement: S10 Fig — (A-C) Immunostaining for cortical layer markers on P7 cortical sections from control and NexCre;β1-Integrinloxp/loxp animals. Staining for Cux1 (A, green, layers II-III) and Ctip2 (A, magenta, layers V and VI), or Rorβ (B, red, layer IV), or Tbr1 (C, red, layer VI), are shown. Sections were counterstained with DAPI (blue). n = 3 animals per genotype for each marker. Scale bars: 500 μm. (PDF) [file pbio.3002212.s010.pdf]

# RGC E13.5 4DIV

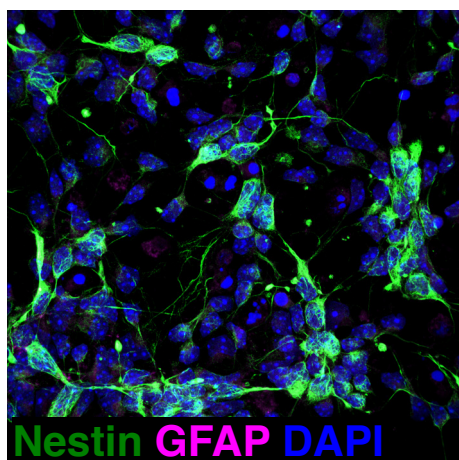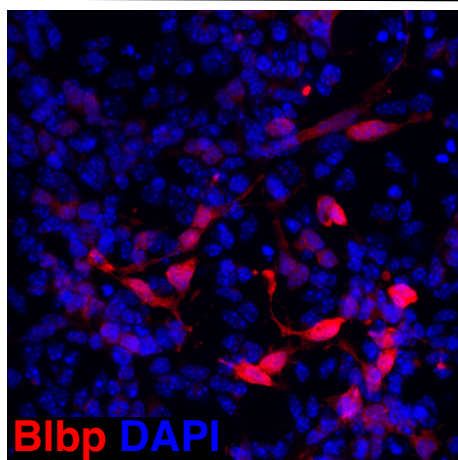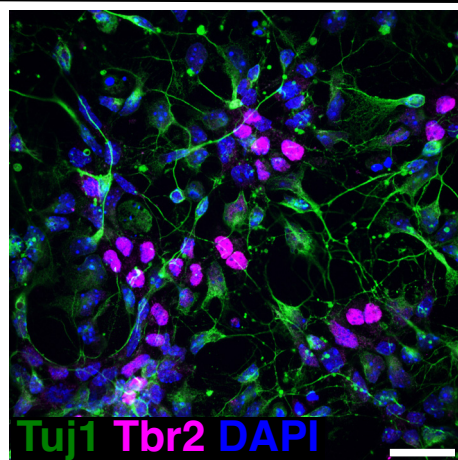

Supplement: S11 Fig — Example of E13.5 4DIV (four days in vitro) mixed primary cortical cultures after staining for neural markers to identify distinct cell types. Left panel: Nestin (green, neuroepithelial and radial glial cells), GFAP (magenta, astrocytes); middle panel: Blbp (red, RGCs); right panel: Tuj1 (green, postmitotic neurons), Tbr2 (magenta, intermediate progenitors). DAPI was used to counterstain nuclei (blue). n = 3 independent cultures. Scale bar: 25 μm. (PDF) [file pbio.3002212.s011.pdf]
