# Supplementary material for: An adhesion signaling axis involving Dystroglycan, β1-Integrin, and Cas adaptor proteins regulates the establishment of the cortical glial scaffold
Source: PLoS Biol. 2023 Aug 4;21(8):e3002212. doi: 10.1371/journal.pbio.3002212 (PMC10431685; doi:10.1371/journal.pbio.3002212)
Supplement: S4 Fig — Coronal sections of P7 Emx1Cre;p130Casflox/+;CasL+/−;Sin+/−, Emx1Cre; p130Casflox/+;CasL−/−;Sin−/−, Emx1Cre; p130Casflox/−;CasL+/−;Sin−/−, Emx1Cre; p130Casflox/−;CasL−/−;Sin+/−, and Emx1Cre; p130Casflox/−;CasL−/−;Sin−/− (Emx1Cre;CasTcKO) cortices stained for the layer markers Ctip2 (green, layers V and VI) and Tbr1 (red, Layer VI), and the mature neuron marker NeuN (grey), counterstained with DAPI (blue). Only Emx1Cre; p130Casflox/−;CasL−/−;Sin+/− and Emx1Cre; p130Casflox/−;CasL−/−;Sin−/− showed the cobblestone cortex phenotype. Bottom right panel: quantification of the # of dysplasia/mm2 in the different transgenic animals. Values given are mean ± SEM, n = 3–8 independent samples per group, 3 sections per sample, Mann–Whitney U test and Bonferroni correction, *p < 0.005 vs. control; ** p < 0.0005 vs. control. For data plotted in graphs, see S3 Data. Scale bar: 1 mm. (PDF) [file pbio.3002212.s004.pdf]

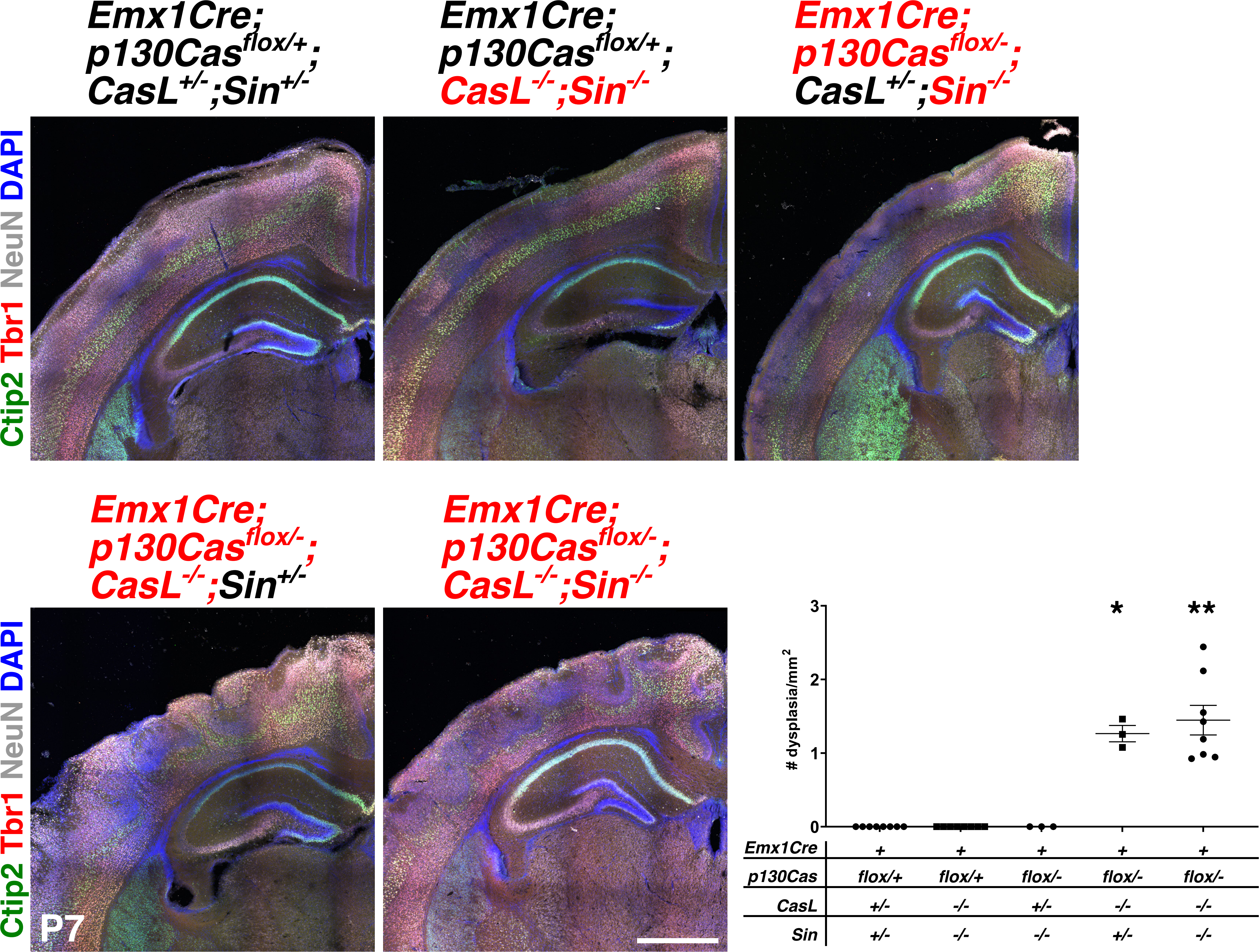
Emx1Cre; p130Cas flox/+; CasL+/-;Sin+/-
Emx1Cre; p130Cas flox/+; CasL-/-;Sin-/-
Emx1Cre; p130Cas flox/-; CasL+/-;Sin-/-
Emx1Cre; p130Cas flox/-; CasL-/-;Sin+/-
Emx1Cre; p130Cas flox/-; CasL-/-;Sin-/-
Ctip2 Tbr1 NeuN DAPI
Ctip2 Tbr1 NeuN DAPI
P7
dysplasia/mm2
0
1
2
3
*
**
Emx1Cre + + + + +
p130Cas flox/+ flox/+ flox/- flox/- flox/-
CasL +/- -/- +/- -/- -/-
Sin +/- -/- -/- +/- -/-
