# Supplementary material for: An adhesion signaling axis involving Dystroglycan, β1-Integrin, and Cas adaptor proteins regulates the establishment of the cortical glial scaffold
Source: PLoS Biol. 2023 Aug 4;21(8):e3002212. doi: 10.1371/journal.pbio.3002212 (PMC10431685; doi:10.1371/journal.pbio.3002212)
Supplement: S12 Fig — Immunostaining of P0 control and Emx1Cre;CasTcKO coronal sections of the contralateral hemispheres to the sides coelectroporated with ΔSrc + p130Cas-ZipB (negative control) or Src-ZipA + p130Cas-ZipB (FIT rescue). Sections were stained for the layers V and VI marker Ctip2 (green) and Laminin (magenta), and counterstained with DAPI (blue). In utero electroporation of one side does not affect the expected phenotype on the opposite hemisphere: i.e., cobblestone phenotypes are still observed in the contralateral side of ΔSrc + p130Cas-ZipB or Src-ZipA + p130Cas-ZipB electroporated Emx1Cre;CasTcKO cortices. n = 6 animals per genotype. Scale bar: 250 μm. (PDF) [file pbio.3002212.s012.pdf]

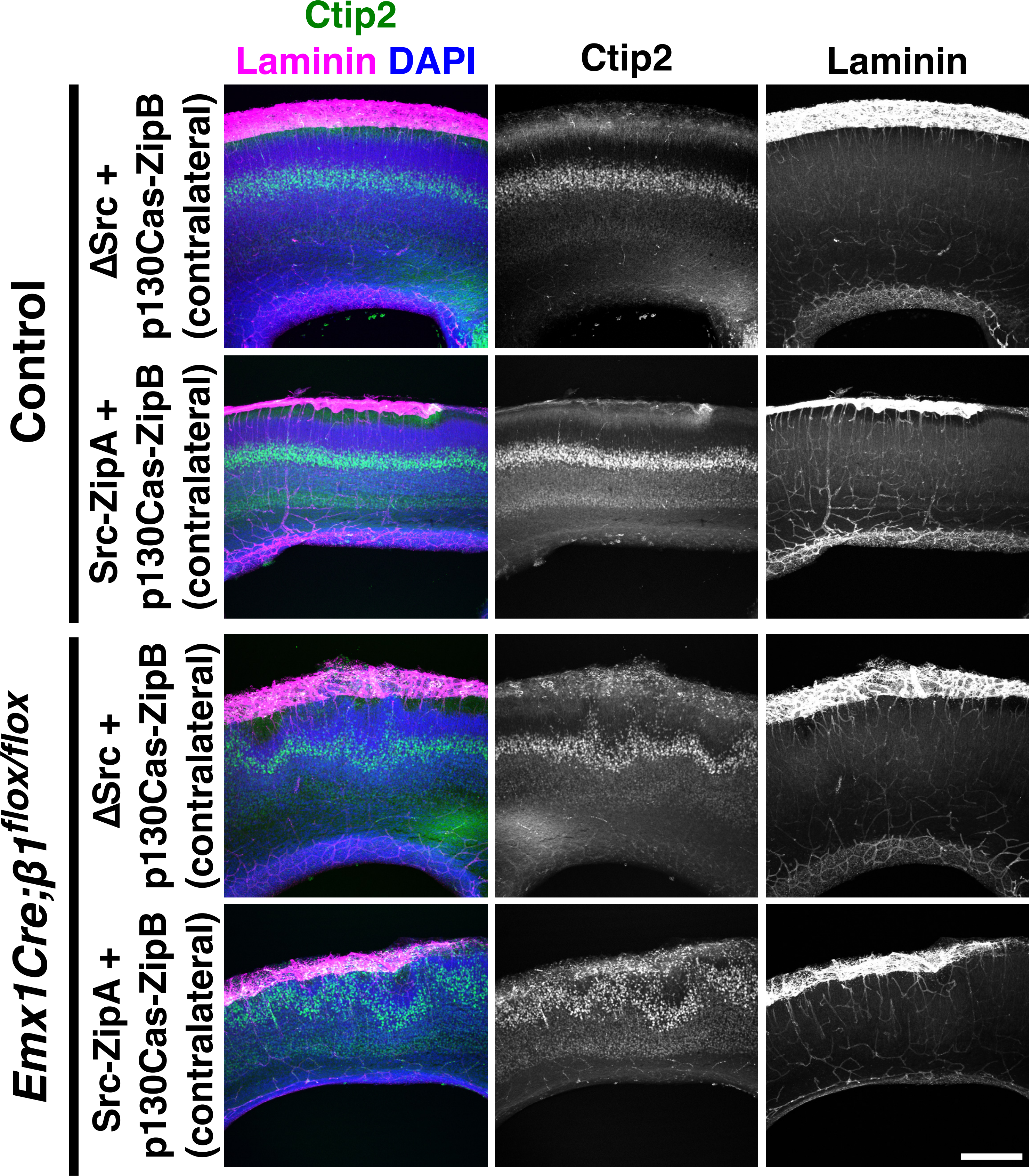

Ctip2
Laminin DAPI
Ctip2
Laminin
Control
ΔSrc + p130Cas-ZipB (contralateral)
Src-ZipA + p130Cas-ZipB (contralateral)
Emx1Cre;β1flox/flox
ΔSrc + p130Cas-ZipB (contralateral)
Src-ZipA + p130Cas-ZipB (contralateral)
